# Older Age, Steroid Exposure, and Digital Pitting Scars Predict Poor Outcomes in Scleroderma Renal Crisis: A 30-Year Retrospective Cohort Study

**DOI:** 10.31138/mjr.140725.epr

**Published:** 2026-06-01

**Authors:** Abhishek Gollarahalli Patel, Sakir Ahmed, Narayan Prasad, Vikas Agarwal

**Affiliations:** 1 Department of Clinical Immunology and Rheumatology, Sanjay Gandhi Postgraduate Institute of Medical Sciences, Lucknow, India;; 2 Department of Clinical Immunology and Rheumatology, King George Medical University, Lucknow, India;; 3 Department of Clinical Immunology and Rheumatology, Kalinga Institute of Medical Sciences, Bhubaneswar, Odisha, India;; 4 Department of Nephrology, Sanjay Gandhi Postgraduate Institute of Medical Sciences, Lucknow, India

**Keywords:** renal insufficiency, scleroderma systemic, signs and symptoms, retrospective studies, risk factors, steroids

## Abstract

**Background:**

Scleroderma renal crisis (SRC) is a rare but severe complication of systemic sclerosis (SSc), often associated with high morbidity and mortality. Data from Indian population remains limited.

**Objectives:**

To describe the clinical features, predictors, and outcomes of SRC in a large, single-centre Indian SSc cohort over three decades.

**Methods:**

This retrospective cohort study included 880 SSc patients diagnosed between 1990 and 2019, classified by ARA 1980 or ACR/EULAR 2013 criteria. SRC was defined by new-onset hypertension and/or rapidly progressive renal failure, with supportive hematologic and urinary findings. Demographic, clinical, and serologic variables were analysed. Predictors of SRC were identified using multivariate logistic regression, and survival was assessed using Kaplan-Meier and Cox regression analyses.

**Results:**

SRC occurred in 27 patients (3.0%), with 85.2% developing SRC within one year of diagnosis. Steroid use preceded SRC in 66.6% of cases. SRC was associated with older age (OR 1.03, 95% CI 1.001–1.072), digital pitting scars (OR 6.16, 95% CI 1.60–23.65), and reduced by immunosuppressive therapy (OR 0.39, 95% CI 0.16–0.98). SRC patients had a significantly higher mortality risk (HR 3.66, 95% CI 1.94–6.89) and shorter survival (mean 7.2 vs. 23.8 years). Dialysis was required in 51.8% of SRC cases.

**Conclusions:**

SRC affected 3% of SSc patients and was associated with high mortality and dialysis dependence. Older age, steroid exposure, and digital pitting scars were key risk factors, while immunosuppression appeared protective. These findings highlight the importance of early identification and careful therapeutic strategies in high-risk patients.

## INTRODUCTION

Systemic sclerosis (SSc) is a chronic autoimmune disease characterised by endothelial dysfunction, vascular abnormalities, and progressive fibrosis affecting the skin and internal organs. It is also associated with a spectrum of pathogenic autoantibodies that contribute to its clinical heterogeneity and disease progression.^1,2^ The prevalence of SSc ranges from 200 to 260 cases per million in the United States and Australia,^3,4^ while in Asia, the prevalence is notably lower, ranging between 20 and 50 cases per million.^5^ Among rheumatic diseases, SSc exhibits the highest standardised mortality ratio (SMR), reflecting a significantly elevated risk of death compared to the general population.^6^ A meta-analysis up to 2006 estimated the SMR in SSc patients to be 3.53.^7^

Scleroderma renal crisis (SRC) is an uncommon but potentially life-threatening complication of SSc. According to data from the EUSTAR cohort, SRC occurs in fewer than 5% of patients with diffuse cutaneous SSc (dSSc) and in less than 2% of those with limited cutaneous SSc (lSSc).^8^ Although rare, SRC has been reported even in patients with sine scleroderma.^9^ It typically develops within the first five years of SSc onset,^10^ with African American patients exhibiting a higher risk.^11^ Despite advances in the understanding and treatment of SSc, SRC remains a clinical challenge due to its rapid progression and association with high morbidity and mortality.

This study aims to provide detailed insights into the clinical presentation, laboratory findings, and long-term outcomes of SRC based on a single-centre cohort (COSSIL-Cohort of Systemic Sclerosis in Lucknow) in Lucknow, India, covering a span of more than three decades. Although extensive research has been conducted globally on SSc and SRC, data from Indian populations remain limited. By analysing the disease characteristics and outcomes in this demographic, our study fills an important knowledge gap and may uncover region-specific patterns distinct from those observed in Western and other Asian cohorts. These findings have the potential to inform clinical practice and improve management strategies in similar epidemiological settings.

## METHODS

We retrospectively reviewed medical records of patients diagnosed with SSc at our centre between 1990 and December 2019. SSc diagnosis was based on the 1980 American Rheumatism Association (ARA) criteria^12^ or the 2013 American College of Rheumatology/European League Against Rheumatism (ACR/EULAR) classification criteria.^13^ We have excluded patients with overlap syndrome to have a homogenous cohort of patients. Data were collected from outpatient and inpatient records, including paper files and electronic health records maintained by the treating physician or fellow-in-training.

Patients were followed up at intervals of two to three months. Data recorded included clinical manifestations, laboratory investigations (complete blood count, renal and liver function tests, pulmonary function tests), and imaging findings where applicable. Baseline information included demographics, organ involvement, and disease extent, evaluated by modified Rodnan skin score (mRSS) and pulmonary function parameters. Data on immunosuppressive therapy, complications including SRC, and outcomes were documented. Clinical, laboratory, and therapeutic variables were compared between patients with and without SRC to identify predictive factors.

SRC was diagnosed by the treating rheumatologist based on the presence of new-onset hypertension and/or rapidly progressive renal failure, with or without >2% schistocytes in peripheral smear, along with supportive findings such as anaemia, thrombocytopenia, and proteinuria.^14^ Thrombotic microangiopathy (TMA) was defined as thrombocytopenia (<100,000/mm^3^) or elevated reticulocyte counts.^15^ In-hospital mortality was retrieved from electronic medical records, while outpatient deaths were reported by family members and documented in clinic files.

### Ethics

Given the retrospective nature of the study, the Institutional Ethics Committee reviewed and approved the protocol and waived the requirement for informed consent. This decision was based on the utilisation of data from clinical records and electronic health systems collected over more than three decades without direct patient interaction. The ethics approval was granted under protocol number PGI/BE/129/2020 dated 21^st^ February.

### Statistical Analysis

Baseline characteristics were summarised as medians with interquartile ranges (IQR) for continuous variables and percentages for categorical variables. Continuous variables were compared using the student’s t-test or binary regression, while categorical variables were assessed with the Chi-square test or Fisher’s exact test, as appropriate.

Univariate analysis was first performed to examine the association of individual clinical and laboratory variables with the occurrence of SRC. Variables showing a p-value <0.20 in univariate analysis were then selected for inclusion in a multivariate logistic regression model. This approach allowed for the identification of independent predictors of SRC after adjusting for potential confounders. Kaplan-Meier survival curves were generated to compare survival between SRC and non-SRC groups, with statistical significance evaluated using the log-rank test. Cox proportional hazards regression was employed to estimate hazard ratios for mortality. All analyses were performed using IBM SPSS software, version 26.

## RESULTS

### Demographics

A total of 880 patients were included, with a female-to-male ratio of 7.7:1 (779 females and 101 males). The mean age at diagnosis was 35.5 ± 12 years (range: 6 to 70 years). The median symptom duration before diagnosis was 24 months (IQR: 12–60 months). The cumulative follow-up amounted to 3,029.33 person-years, with a median follow-up duration of 471.5 days (IQR: 28–1,830 days).

Diffuse cutaneous SSc was the predominant subtype, seen in 517 patients (58.8%), followed by limited cutaneous SSc in 283 (32.1%) and sine scleroderma in 80 (9.1%).

### Clinical Features

Cutaneous involvement was the most common manifestation, with a median mRSS of 16 (IQR: 7–26). Severe skin thickening (mRSS >40) was observed in 43 patients. Raynaud’s phenomenon was the most frequent clinical sign, present in 90.9% of patients. Digital ulcers and pitting scars were found in 50.6% and 38.9% of patients, respectively, and 9% developed digital gangrene.

Inflammatory arthritis, primarily involving small joints, was reported in 31% of patients. Myositis occurred in 10.5% of patients, and flexion contractures in 11.1%. Gastrointestinal symptoms, including dyspepsia, bloating, and anorexia, were present in 57.3%. Male patients more often presented with diffuse skin involvement and had shorter symptom duration before diagnosis. Interstitial lung disease (ILD) was identified in 51.4% of patients, and pulmonary arterial hypertension (PAH) in 19.9%. Cardiac involvement was documented in 51 patients. A total of 233 infections were reported among 153 patients, most commonly involving the lungs (46.3%) and skin/soft tissue (21.8%). Tuberculosis accounted for one-third of pulmonary infections. Among 90 reported deaths, more than one-third were due to lung complications, including progressive ILD (13 deaths) and lower respiratory tract infections (19 deaths).

### Autoantibodies

Among the 654 patients tested, antinuclear antibodies (ANA) were positive in 91.2%. The most common ANA patterns were homogeneous (41.8%), speckled (34.2%), and nucleolar (11%). Anti-Scl-70 was the most frequent extractable nuclear antigen (ENA) antibody (59%), followed by anti-Ro (13.8%), anti-RNP (12.8%), and anti-centromere (4%).

### Scleroderma Renal Crisis

SRC was identified in 27 patients (3.0%). Of these, 85.2% (23/27) developed SRC within one year of diagnosis. Steroid use within six months prior to SRC was noted in 66.6% of patients. At presentation, 85.2% were hypertensive, 92.6% had renal dysfunction, and 51.8% had urinary abnormalities, such as proteinuria or haematuria. During follow-up, 11 SRC patients died, with six deaths directly attributed to SRC, and eight progressed to chronic kidney disease (**Table 1**).

**Table 1.** Comparison of demographics, clinical features, and outcomes of patients with or without SRC.

**Characteristic**	**No SRC (n=853)**	**SRC (n=27)**	**Odds ratio**
Age (years)*	35.3(11.9)	41.9(12.4)	1.04(1.01–1.07)
Sex (Females)	756	23	0.73(0.25–2.17
Total admissions^	0 (0 to 23 )	2 (0 to 11)	
Disease admissions^	0 (0 to 13)	1 (0 to 11)	
Follow up duration(months)#	40.3(36.5–44.2)	49.2(30.5–67.9)	
Symptoms duration (months)#	39.7(37.3–42.1)	34.9(22.0–47.7)	
mRSS*	17.5(12.3)	15.4(13.5)	0.98(0.95–1.01)
Raynaud phenomenon	777	23	0.56(0.25–1.24)
Digital ulcers	435	10	0.56(0.25–1.24)
Digital pitting scars	339	3	0.19(0.05–0.63)
Joint contractures	94	4	1.40(0.47—4.14
Calcinosis	36	0	0.00(0.00–0.00 )
Gangrene	78	1	0.38(0.05–2.85)
GERD	487	17	1.27(0.57–2.82)
Arthritis	264	9	1.11(0.49–2.51)
Myositis	86	6	2.54(1.00–6.48)
Sicca symptoms	34	1	0.92(0.12–7.03)
ILD	433	19	2.3(0.99–5.32)
PAH	166	9	2.06(0.91–4.68)
Cardiac involvement	48	3	2.09(0.61–7.20)
Infection number	0.25(0.20–0.29)	0.85(0.40–1.3)	
Immunosuppression	193	14	3.68(1.70–7.96)
Infection ever	140	13	4.72(2.17–10.27)

* Age and mRSS: mean and SD.

^ Total and disease admissions: median and range.

# Follow-up duration, symptom duration, infection number: Mean and 95% CI.

mRSS: modified Rodnan skin score; GERD: Gastroesophageal reflux disease; ILD: Interstitial Lung disease; PAH: Pulmonary arterial hypertension; SRC: Scleroderma renal crisis.

Patients with SRC were significantly older than those without SRC (mean age: 41.9 vs. 35.3 years; OR 1.04, 95% CI: 1.01–1.07). This association remained significant in multivariate analysis (OR: 1.03, 95% CI: 1.001–1.072). Digital pitting scars were associated with a decreased risk in univariate analysis (OR: 0.19, 95% CI: 0.05–0.63), but in multivariate analysis, they were significantly associated with SRC (OR: 6.16, 95% CI: 1.60–23.65) (**Table 2**).

**Table 2.** Univariate and multivariate analysis for the risk of scleroderma renal crisis.

	**Univariate analysis**		**Multivariate analysis**	
**Variables**	**OR**	**95% CI**	**OR**	**95% CI**
Age (years)	1.04	1.01–1.07	**1.03**	**1.001–1.072**
Total admissions			0.95	0.77–1.17
Disease admissions			1.21	0.85–1.70
Digital ulcers	0.56	0.25–1.24	0.98	0.40–2.41
Digital pitting scars	0.19	0.05–0.63	**6.16**	**1.60–23.65**
Myositis	2.54	1.00–6.48	0.64	0.22–1.81
ILD	2.3	0.99–5.32	0.80	0.31–2.09
PAH	2.06	0.91–4.68	0.91	0.35–2.35
Infection number			1.32	0.65–2.68
Immunosuppression	3.68	1.70–7.96	**0.39**	**0.16–0.98**
Infection ever	4.72	2.17–10.27	0.57	0.14–2.33

ILD: Interstitial lung disease; PAH: Pulmonary arterial hypertension.

Immunosuppressive therapy was associated with increased SRC risk in univariate analysis (OR: 3.68, 95% CI: 1.70–7.96), but showed a protective effect in multivariate analysis (OR: 0.39, 95% CI: 0.16–0.98). A history of infection predicted SRC in univariate analysis (OR: 4.72, 95% CI: 2.17–10.27), but not in multivariate analysis.

Other variables, such as digital ulcers, myositis, ILD, PAH, and infection number, were not significantly associated with SRC in multivariate models, despite some showing univariate significance (**Table 2**).

**Figure 1.**
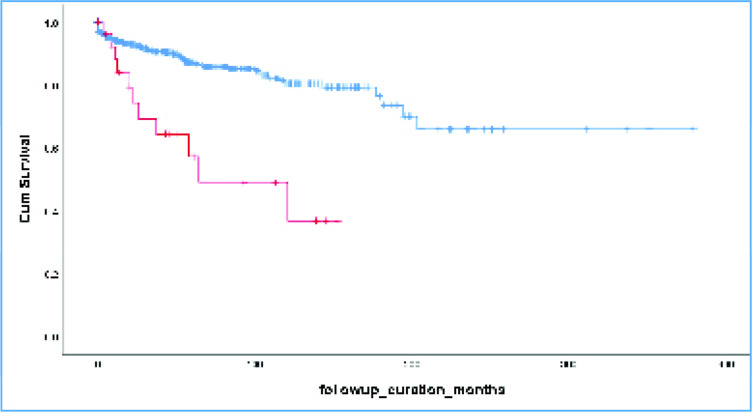
Kaplan Meier survival analysis for scleroderma renal crisis (SRC) vs Non-SRC; Red: SRC, Blue: Non-SRC.

Survival analysis revealed a significantly increased risk of mortality in SRC patients (HR: 3.66, 95% CI: 1.94–6.89) (**Figure 1**). The mean survival time for SRC patients was 7.2 years (95% CI: 4.9–9.5), compared to 23.8 years (95% CI: 21.5–26.0) for non-SRC patients (**Figure 1**).

Because the use of immunosuppressive therapy in systemic sclerosis has changed over the years, we performed a subgroup analysis stratifying patients by year of diagnosis (before vs. after 2010). We chose 2010 as a cutoff, as we could find a statistically significant difference in the use of immunosuppression before and after 2010. Among 395 patients diagnosed before 2010, 9 developed SRC, compared to 18 cases among 485 patients diagnosed after 2010. Although the absolute number of SRC cases was higher in the post-2010 group, this difference was not statistically significant (Relative Risk: 0.60; 95% CI: 0.27–1.36).

## DISCUSSION

Our study provides a comprehensive analysis of a large cohort of SSc patients, shedding light on the clinical and demographic factors associated with SRC, as well as its impact on survival.

Patients with SRC were notably older than those without SRC, consistent with findings reported in various other studies.^14,16,17^ Each additional year of age increased the odds of SRC by 4%. Digital pitting scars were initially linked to decreased odds of SRC in univariate analysis, but in multivariate analysis, they were associated with a significantly increased risk of SRC. This dramatic change suggests that digital pitting scars might be a complex marker of disease severity or progression, where their significance becomes apparent only after adjusting for other variables. It is possible that digital pitting scars are a proxy for more severe underlying vascular disease, which could pre-dispose patients to SRC. Immunosuppressive therapy was initially linked to a higher risk of SRC in univariate analysis, but in multivariate analysis, it showed a protective effect against SRC. This finding suggests that while patients receiving immunosuppression may initially appear to be at higher risk for SRC, possibly due to more severe disease, the therapy itself might help mitigate the risk when adjusted for other factors. This protective effect warrants further investigation to clarify the role of immunosuppressive treatment in preventing SRC. Patients with SRC had a significantly higher median number of disease-specific admissions compared to those without SRC, possibly indicating that severe or frequent manifestations are at a higher risk of developing SRC, emphasising the importance of close monitoring in these individuals, though these factors didn’t remain statistically significant in multivariate analysis. We didn’t find an association with cardiac involvement, which has been noted in studies like that of Demarco et al., and Guillevin et al.^15,18,19^ Other features, which were associated factors of SRC in other studies, including digital gangrene, arthralgia, baseline mRSS, and large joint contractures, were not associated with SRC in our study.^15,18,19^ A case report has documented the rare occurrence of SRC and SSc-PAH developing simultaneously in a patient with limited scleroderma.^20^ Another study highlighted several established SRC risk factors, including dcSSc subset, large joint contractures, cardiac enlargement, low DLCO, pulmonary hypertension, short disease duration, and possible cardiomyopathy, with the close temporal link to corticosteroid use, particularly in one patient without recent-onset SSc, suggesting steroids may play a predominant triggering role.^21^ Although the occurrence of SRC appeared numerically higher in the post-2010 group, this difference was not statistically significant, which may reflect the overall low number of SRC events rather than a true absence of effect of evolving treatment practices.

We compared patient demographics and clinical features of various studies on SRC from different countries and time periods to provide insights into the global landscape of SRC (**Table 3**). The prevalence of SRC varied widely, ranging from 1.74% in the EUSTAR study^22^ to as high as 20% in the study from Thailand.^19^

**Table 3.** Patient demographics and clinical features across various cohorts across the globe.

**Patient characteristics**	**Penn et al.** ^14^	**Loic Guillevin et al.** ^15^	**ISRCS USA n(%)** ^22^	**Moinzadeh, et al.** ^19^	**EUSTAR** ^20^	**Our Study**
**Country**	UK	France	Canada	Thailand	Europe	India
**Year of study**	2007	2011	2014	2017	2020	2025
**Study Design**	Retrospective	Retrospective	Prospective	Retrospective	Prospective	Retrospective
**Number of patients**	110	91	75	19	169	27
**SRC prevalence**	5%	17.5%		20%	1.74%	3.0%
**Ethnicity**	White	White	White	Asian	White	Asian
**Age^a^**	50.7^b^ (24 –80)	50(15)	52.5(12.2)	56(13.8)	57.3^b^(48–67.9)	41.9(12.4)
**Female(%)**	81	75.8	66.6	52.6	76.3	85.1
**dSSc(%)**	78	85.7	74.7	89.5	44	62.9
**Disease duration^b^** **(in years)**	0.6(0–16.6)	NA	1.5(0.9 to3.7)	NA	5 (1.9–10.9)	2.9(1.8–3.9)
**GC prior use(%)**	59	70.3	44	52.6	41	66.6
**NSAID prior use (%)**	NA	NA	14.7	NA	NA	22.2
**Recent cardiac(%)**	NA	46.2	17.3	52.6	NA	18.5
**Anaemia(%)**	52		37.3	NA	NA	96.2
**Normotensive(%)**	NA	22	6.65	36.8	NA	14.8
**Death by 2 years(%)**	26	40.7	36	52.6	NA	44.4
**Any dialysis(%)**	64	53.8	53	78.8	NA	51.8
**CKD(%)**	41	39.6	40	31.5	NA	29.6
**Death due to SRC(%)**	NA	12/37	NA	NA	NA	18.5
**Scl 70(%)**	17.2	31	25.3	67.2%	45.5	51.8
**RNAP III(%)**	59	5.5	20	NA	NA	NA
**Speckled(%)**	60		25.3	NA	NA	29.6
**Haemolysis(%)**	52	61.5	NA	52.6	NA	18.5
**TMA(%)**	59	56	NA	47.4	NA	29.6
**Proteinuria(%)**	NA	68.5	NA	63.2	NA	51.8
**Haematuria(%)**	NA	41.5	NA	31.6	NA	NA

a Mean (Standard Deviation);

b Median (Interquartile range).

CKD: Chronic kidney disease; EUSTAR: European Scleroderma Trials And Research group; ISRCS: International Scleroderma Renal Crisis Survey; NSAID: Nonsteroidal anti-inflammatory drug; GC: Glucocorticoid; RNAP III: Ribonucleic acid polymerase III; SRC: Scleroderma renal crisis; TMA: Thrombotic microangiopathy.

Our study reported a prevalence of 3.0%. This variation could be attributed to differences in study populations, diagnostic criteria, and healthcare access. The average age of SRC patients in our study (41.9 years) was notably younger compared to other cohorts, like the EUSTAR study (57.3 years)^22^ or the study by Penn et al. (50.7 years).^14^ Our cohort also had a higher percentage of females (85.1%) compared to most other studies.^23^ The majority of patients in our study had dSSc (62.9%), which is higher than in the EUSTAR study (44%) but lower than in the Thai cohort (89.5%). In a UK study, the likelihood of developing SRC was significantly higher in patients with dcSSc compared to those with lcSSc, with an OR of 7.2 (95% CI: 4.5–11.4).^14^ Our patients developed SRC after a median of 2.9 years of disease, whereas the EUSTAR cohort reported a median of 5 years. The relatively shorter duration in our cohort could be attributable to the higher proportion of diffuse cutaneous SSc, which carries a greater risk of early SRC. Most patients developed SRC in the first year after diagnosis (89.2%), whereas 66% of patients in a UK study experienced SRC within the same time frame following their scleroderma diagnosis.^14^ The use of glucocorticoids (GC) prior to SRC was high across studies, with our study reporting a usage rate of 66.6%. This aligns with the understanding that GC use may be a risk factor for SRC. The use of NSAIDs was reported only in our study (22.2%) and the Canadian cohort (14.7%).^24^ Our study reported a strikingly high rate of anaemia (96.2%) compared to other studies, suggesting that anaemia is a prevalent and possibly under-recognised feature of SRC in our population, or it could be secondary to malnutrition linked to low per capita income. The percentage of patients with normotensive SRC in our study (14.8%) was higher than in the Canadian cohort (6.65%) but lower than in the Thai cohort (36.8%). The rates of haemolysis (18.5%) and thrombotic microangiopathy (TMA) (29.6%) in our study were lower than in Western cohorts.

We used ACE inhibitors in all patients for the management of SRC. In a study to evaluate whether the use of ACE inhibitors before the onset of SRC affects the outcomes, it was seen that prior exposure was associated with an increased risk of death (hazard ratio 2.17, 95% CI 0.88–5.33) after adjusting for pre-existing hypertension.^24^ A similar study from EUSTAR cohort had HR of 2.04 (95%CI 1.29–3.24).^22^ The EULAR guidelines emphasise that current evidence does not support the use of ACE inhibitors as a preventive measure to reduce the risk of developing SRC or to improve its outcomes.^25^ Prophylactic ACE inhibitor treatment may be harmful due to its potential to mask hypertension, which could delay the diagnosis of SRC, or the phenomenon of aldosterone breakthrough in patients on long-term ACE inhibitor therapy.^26^ More than half of our patients were on calcium channel blockers (CCB) (55.5%). The use of calcium channel blockers (CCBs), angiotensin receptor blockers (ARBs), endothelin receptor antagonists, and glucocorticoids under 15 mg of prednisolone did not affect the risk of developing SRC in EUSTAR analysis.^22^ The presence of Scl-70 antibodies in our study (51.8%) was higher than in most other studies, suggesting a potential association with more severe disease or a higher risk of SRC in our population. Testing for anti-RNA polymerase was not available at our centre due to logistic reasons. A recent study from the EUSTAR registry, which included more than 2,800 SSc patients, found that anti-RNA polymerase antibodies were independently linked to SRC, with an odds ratio of 5.86 (95% CI 2.6 to 13.2).^27^ The prevalence of anti-RNA polymer-ase antibodies is high in United States^28^ and Sweden,^29^ i.e., 28% and 22% respectively, but is low in Asia, with a prevalence of 6% in Japan^28^ and 3.4% in South Korea.^30^ Mortality within two years of SRC was high across studies, with our study reporting a 44.4% mortality rate. This is comparable to the rates reported by Penn et al. (26%) and the Canadian study (36%) but lower than the Thai study (52.6%). Patients with SRC had a more than threefold increased risk of death compared to those without SRC, with a striking reduction in mean survival time. In the post-ACE inhibitors era, the rate of permanent dialysis following scleroderma renal crisis (SRC) has ranged between 19% and 40%.^31^ The percentage of patients requiring dialysis in our study (51.8%) was like other cohorts, indicating severe renal involvement. Renal recovery after SRC has been reported in up to 25% of patients within 18 months of the crisis.^32^ Between 3% and 17% of patients with SSc eventually undergo renal transplantation. Survival rates are higher in patients who receive a renal transplant, ranging from 54% to 91%, compared to those remaining on dialysis, where survival rates range from 31% to 56%.

The strengths of our study include its large sample size and its comprehensive span over more than three decades. Utilising electronic health records allowed us to access extensive data on admissions, reasons for admissions, outcomes, and laboratory parameters for most patients.

Limitations of this study are its retrospective design, which includes gaps in data due to less comprehensive electronic health records before 2000. Detailed information on the cumulative or average steroid dose, as well as recent dose changes prior to SRC onset, was not consistently available in our dataset. Additionally, deaths occurring outside hospital settings may have been related to SRC but were not always documented due to recall bias from relatives providing death information. Furthermore, we did not have access to death certificates from practitioners, hospitals, or state/national authorities. Another limitation is the non-availability of testing for RNA polymerase III antibody, the association of which could not be assessed.

## CONCLUSIONS

Scleroderma renal crisis was identified in 3% of our systemic sclerosis cohort and was associated with a 40.7% mortality rate during follow-up. Steroid use, older age, and digital pitting scars were significant risk factors, while immunosuppressive therapy appeared protective. The younger age at onset, higher female predominance, and high anaemia prevalence may reflect population-specific disease features. Early recognition and timely management of SRC remain critical to improving outcomes.

### Future Perspectives and Research Agenda

To build on our findings, future research should focus on prospective, large-scale studies to validate identified risk factors and refine SRC risk prediction models. Longitudinal studies assessing treatment response and long-term outcomes are warranted. Research into novel biomarkers, including underexplored autoantibodies, may enhance early SRC detection and person-alised care. Additionally, studies evaluating the efficacy and long-term impact of renal transplantation in SRC patients are needed to inform treatment strategies and guide patient counselling.

## Data Availability

Data pertaining to this study shall be shared on reasonable request to the corresponding author (Vikas Agarwal, vikasagr@yahoo.com).

## STATEMENTS AND DECLARATIONS

Ethical considerations: Waiver of written informed consent for retrospective data retrieval was obtained from the Institute Ethics Committee, SGPGIMS, Lucknow, letter number PGI/BE/129/2020, date of approval 21-Feb-2020
